# Poor nutrition for under-five children from poor households in Ethiopia: Evidence from 2016 Demographic and Health Survey

**DOI:** 10.1371/journal.pone.0225996

**Published:** 2019-12-20

**Authors:** Habtamu Kebebe Kasaye, Firew Tekle Bobo, Mekdes Tigistu Yilma, Mirkuzie Woldie

**Affiliations:** 1 Department of Midwifery, Institute of Health Sciences, Wollega University; ekmete, Ethiopia; 2 Department of Public Health, Institute of Health Sciences, Wollega University; Nekmete, Ethiopia; 3 Australian Centre for Public and Population Health Research, Faculty of Health, University of Technology Sydney, Sydney, New South Wales, Australia; 4 Department of Health Policy and Management, Jimma University; Jimma, Ethiopia; 5 Fenot Project of Harvard T.H. Chan School of Public Health, Addis Ababa, Ethiopia; Sefako Makgatho Health Sciences University, SOUTH AFRICA

## Abstract

**Background:**

Ethiopia is commonly affected by drought and famine, and this has taken quite a toll on citizens of the country, particularly the under-five children. Undernutrition among under-five children in Ethiopia is a prominent public health concern, and it lacked attention for decades. However, the government of Ethiopia, together with other stakeholders, committed to overcoming the impact of malnutrition through the transformational plan. Here we show the magnitude of undernutrition among under-five children and the factors predicting the achievement of global nutrition targets set for 2025 at the World Health Assembly.

**Methods:**

Ethiopian Demographic and Health Survey (EDHS) 2016 was used for this study. A total of 9494 child-mother pairs were included in this analysis. The nutritional status indicators (Height-for-age, Weight-for-height and Weight-for-age) of children were measured and categorized based on the World Health Organization child growth standards. A multilevel logistic regression model adjusted for clusters and sampling weights were used to identify factors associated with stunting, underweight, and wasting. The independent variables were assessed by calculating the odds ratios with 95% confidence interval (CI).

**Result:**

The prevalence of stunting was 38.3% (95% CI: 36.4% to 40.2%), under-weight 23.3% (95%CI: 21.9% to 24.9%) and wasting 10.1% (95%, CI: 9.1% to 11.2%). Sex of the child (male), children older than 24 months, recent experience of diarrhea, household wealth index (poorest), and administrative regions (Tigray, Amhara and developing regions) had a higher risk of undernutrition. On the other hand, children born from overweight mothers and educated mother (primary, secondary or higher) had a lower risk of undernutrition.

**Conclusion:**

The burden of undernutrition is still considerably high in Ethiopia. Implimentation of strategies and policies that focus on improving the socioeconomic educatiional status of the community need to be sustained. Generally, actions targeted on factors contributing to undernutrition among under-five children demands immediate attention to achieve national and global nutrition target.

## Background

Malnutrition has been identified as a significant public health priority, and ambitious targets were aimed to achieve [1]. Although the number of the children affected by malnutrition cannot merely be summed, more than 200 million under-five children worldwide suffered from all forms of malnutrition in 2018 as reported by UNICEF, WHO and World Bank joint malnutrition estimates [2]. Malnutrition results from the interaction of poor-quality diets, poor-quality health care, environment and personal behaviors that end up either as over-nutrition or undernutrition. Undernutrition is the result of not taking enough total food energy and nutrients, and overall it represents hunger; whereas the earlier has been identified as an emerging global problem related with an increased level of energy intake beyond the body requirements [3]. Undernutrition contributes to the deaths of about three million children every year and continued to threaten the lives of hundreds of millions of children globally [4].

Stunting, wasting, under-weight and micronutrient deficiencies are the indicators of the undernutrition. Stunting refers to a child who is too short for his or her age (HAZ) that occurs as a result of chronic malnutrition. Whereas, wasting is acute malnutrition or recent rapid weight loss and identified when a child is too thin for his or her height(WHZ). The third category of undernutrition is underweight, which accounts for both acute and chronic under-nutrition. It refers to a child who is too low in weight for his or her age (weight-for-age)[5]. These problems commonly occur secondary to the insufficient household food supply, weak childcare practices, and limited access to quality health and sanitation services[6].

Globally, stunting, wasting and underweight among under-five children account for 22.2%, 7.5%, and 13.5% respectively. Africa and Asia bear the lion share of global undernutrition burden. Stunting and underweight decreased by 43.5% and 45.8% from 1990 to 2017 globally. However, the decline is not uniform in all parts of the world. Ninety per cent of the children suffering from undernutrition resides in Asia and Africa. While Asia made encouraging strides in reducing stunting from 47% in 1990 to 24% in 2016, Africa did not show a satisfactory reduction, 42% in 1990 to 33% in 2016. These disproportional reductions could be more challenging to achieve the Sustainable Development Goals aimed to be achieved by the year 2030 [7,8].

In developing countries, malnutrition remains prevalent, and this has a significant effect on the overall development of the countries. Estimated 3.1 million child deaths annually or 45% of all mortality among under-five children were associated with undernutrition as reported by Black and his colleagues[9,10]. The devastating outcomes of undernutrition do not limit in death, the survivors will be disadvantaged both mentally and socially, while it also affects the overall global economy.

Ethiopia is also among the countries where a significant proportion of children suffer from undernutrition, yet it is an unrecognized public health problem. Reviews revealed that the problem is widespread entirely in all regions, and with a range of distributions from 40.0%-43.3%, 30.8%-33.0% and 12.6%-19.0% of children suffering from stunting, underweight and wasting respectively. [11,12].

Childhood undernutrition is the result of multiple factors. The most consistent predictors associated with undernutrition in this region include low educational status of the mother and father, child’s age, sex of child (male), poor household, low birth weight, mother’s age (<20 years), source of drinking water (unimproved), low mother’s BMI, diarrheal episode. Also, breastfeeding duration, multiple births, low birth weight, the absence of toilet facilities in households, infection, sanitation and health services are some of the significant predictors of malnutrition[13,14].

As a result, reducing the prevalence of undernutrition has become the utmost importance for global nutrition targets of the World Health Assembly[15]. Ethiopia also shared the vision of reducing children malnutrition. To monitor the progress towards the goal, we assessed nationally representative data. To the equal priority, the factors hindering the reduction of undernutrition also needs investigation in scientifically appropriate statistical methods. Therefore, here, we present the size and associated factors of undernutrition using in Ethiopia and thereby aid policymakers and healthcare managers with evidence showing areas demanding hard work.

## Method

### Data source

This analysis was based on data from the Ethiopian Demographic and Health Survey (EDHS) 2016conducted by Central Statistical Agency (CSA) of Ethiopia and ICF international conducted the survey. We extracted the required variables for the study from IPUMS DHS [16] All nine regional states and two administrative cities in Ethiopia were included in the survey. Data were collected from January 18, 2016, to June 27, 2016.

**Fig 1:**
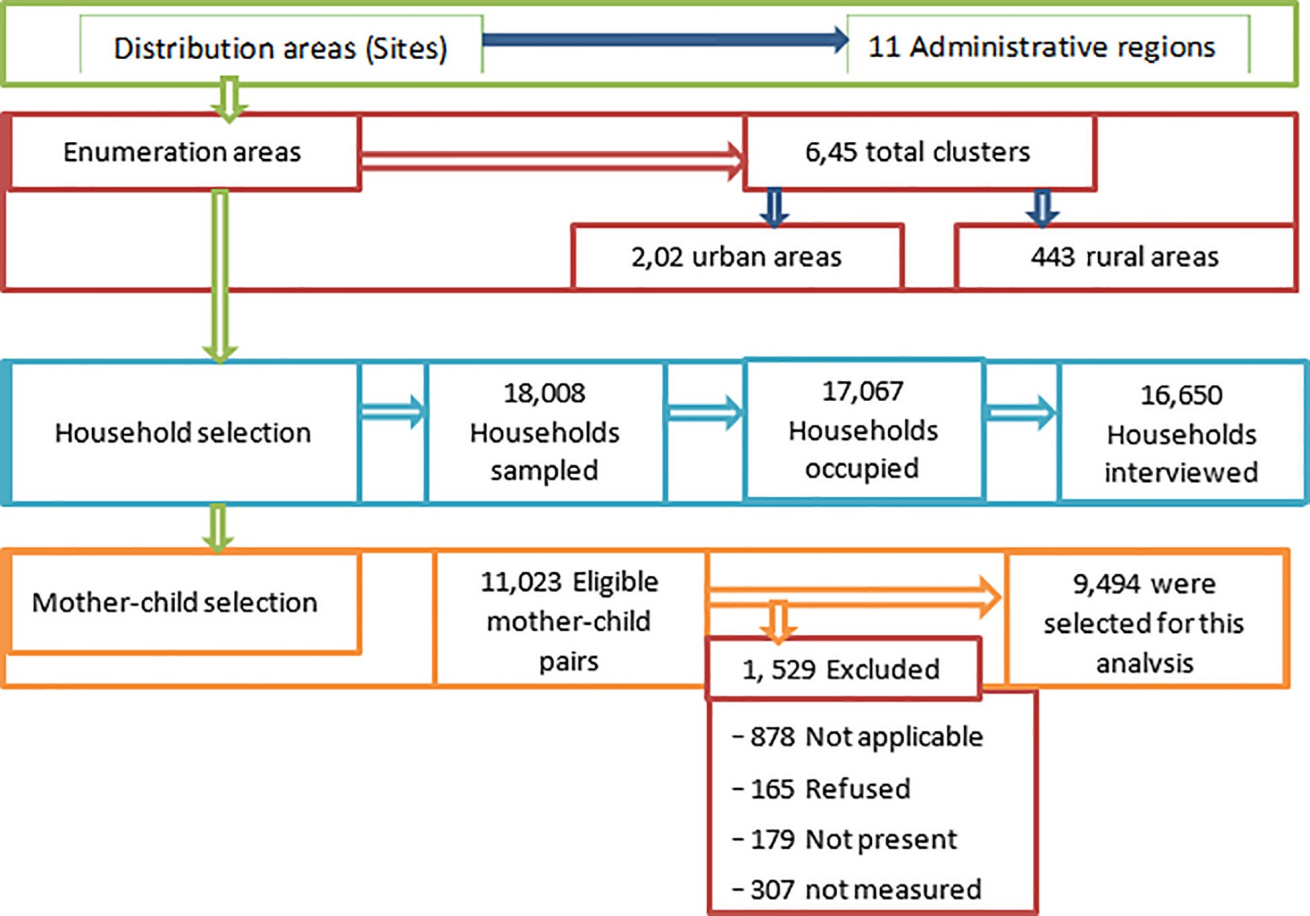
A multiple stage methodology process for selection of mother-child pair at the household level, 2016 EDHS.

The sample was stratified and pulled in two-stages. The sampling frame for the first-stage was based on the Ethiopia Population and Housing Census (PHC) conducted in 2007. In this stage, a total of 645 enumeration areas (EAs) (443 in rural areas and 202 in urban areas) were selected with probability proportional to EA size. In the second stage, using equal probability, systematic selection 28 households were selected from each cluster. For all children younger than five years and their mother’s in the selected household, anthropometric data were collected. In this study we analysed data that the survey team obtained after they screened the children for eligibility critera and excluded some children (12%) from final report because of the misclassification and errors. Finally, 8,757 unweighted (9,464 weighted) mother-child pairs were considered for this study. Fig 1 shows the selection process of the mother-child pair at the household level. The details of the sampling and methodology of the EDHS 2016 were provided in the report of the survey[17].

### Ethics approval and consent to participate

The CSA received the ethical clearance for the survey (EDHS 2016) from Ethiopian Health and Nutrition Research Institute (EHNRI) Review Board, the National Research Ethics Review Committee (NRERC) at the Ministry of Science and Technology, the Institutional Review Board of ICF International, and the CDC. The Central statistical agency obtained written informed consents from the parents of the children for the data obtained from the children, and the permissions for variables regarded with households were granted from the respondents. Voluntary participation was ensured during interviews. We received the survey data from Measure DHS upon submission of a proposal. After data access is authorized from Measure DHS, we have maintained the confidentiality.

### Study variables

#### Outcome variables

The dependent variables were stunting (height-for-age), underweight (weight-for-age), and wasting (weight-for-height). Measurements of height and weight were obtained for under-five children in the selected households. We extracted the raw variable exprecing the standard diviation of each measurement and then we classified the findings to get each undernutrition. Classifications of child undernutrition were made based on WHO Child Growth Standards[18].

Stunting: expressed as a binary outcome, category 0 not stunted if height-for-age Z (HAZ) standard deviations (SD) is greater than −2 and category 1 stunted if Z (HAZ) less than −2SD. Underweight: categorized as 0 no underweight if the weight for age Z (WAZ) is greater than −2 SD and category 1 underweight if Z (WAZ) less than −2SD. Wasting: expressed as category 0 no wasting if the weight-for-height Z (WHZ) is higher than −2 SD and category 1 wasting if Z (WHZ) less than −2SD. We have also reported the prevalence of moderate (between -2SD and -3SD) and severe (less than -3SD) for stunting, underweight and wasting respectively.

#### Explanatory factors

**Fig 2:**
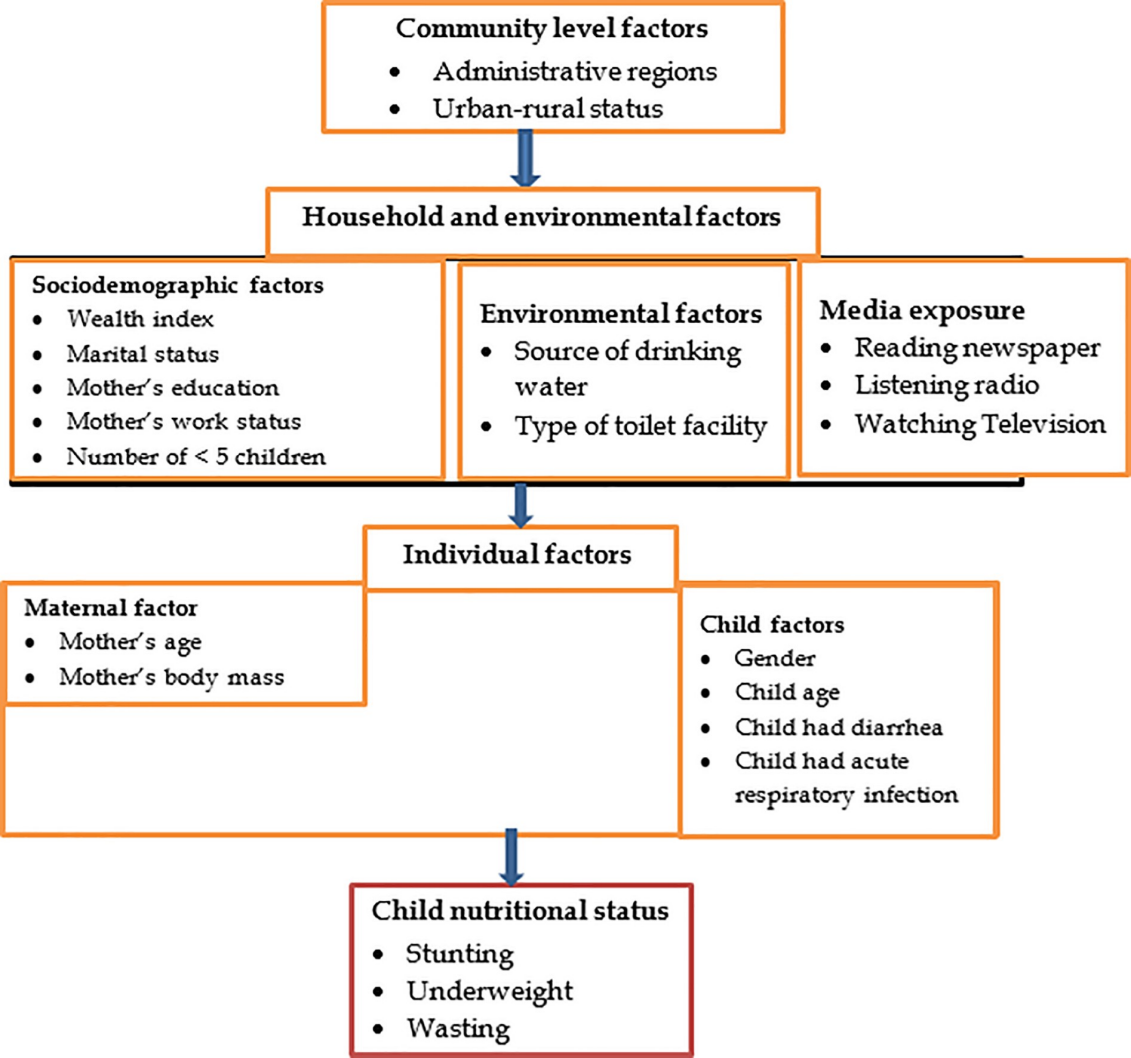
Conceptual framework showing the association between explanatory variables and undernutrition, 2016 EDHS.

The independent variables were identified based on a conceptual framework developed by UNICEF[19] and previous studies in the area of undernutrition among children (Fig 2).

The variables were grouped into three categories which include: community factors, household and environmental factors, and individual factors (Fig 2). Community level factors involve the place of residence (urban-rural status), and administrative regions. The administrative regions were further grouped into six categories based on socioeconomic status and urbanity for this study. Afar, Somali, Benshangul Gumuz, and Gambela are commonly known as developing regional states in Ethiopia because of their lower socioeconomic status and thus grouped. Administrative regions with a predominantly urban population, such as Addis Ababa, Dire Dawa and Harari were also grouped together. However, Oromia, SNNPR, Tigray and Amhara were not grouped.

Household and environmental factors included socio-demographic factors (household wealth index, mother’s education, work status, marital status and the number of under-five children in the house), environmental factors (source of drinking water and type of toilet facility) and media exposure (reading newspaper, listening to the radio and watching television). The individual factors included maternal factors (age and body mass index) and child factors (gender, age, the recent history of diarrhea and acute respiratory infections).

### Statistical analysis

Data analysis was performed after adjusting for sampling design (stratification and clustering) using ‘svy’ command in Stata 14.2. Descriptive characteristics were computed and presented by nutritional status (underweight, stunting and wasting). We reported the prevalence of underweight, stunting and wasting along with confidence interval.

Generalized linear latent and mixed models (gllamm)[20] with the logit link and binomial family that adjusted for clusters and sampling weights were used to identify factors associated with stunting, underweight, and wasting. We first conducted bivariable regressions with each potential risk factor, and the decision to retain the potential risk factor in the final multivariable model was made based on P-value < 0.25. Three multilevel multivariable logistic regression models, one model for each form of undernutrition (stunting, underweight and wasting) were constructed. The adjusted risk of independent variables was assessed by calculating the odds ratios with 95% confidence interval and those with p-value 0.05 were reported from the final model.

## Results

### Descriptive characteristics of study participants

A total of weighted 9494 mother-child pairs were included in the study. Among these, 51.3% were male, and 21.4% were under 12 months. The prevalence of underweight and stunting was higher among male and female children were both relatively equally affected by wasting. Underweight and stunting was less common among children younger than 12 months, compared to other age categories. Majority (30.5%) of the mothers were between the age of 25–29 years, 73.5% had normal body mass index, 65.6% had no education, 89% were from rural, 56.1% had access to improved drinking water source, 54.8% had access to unimproved toilet facility and 43.9% of the study participants were from Oromia (Table 1).

**Table 1:** Characteristic of the study participants by stunting, underweight, and wasting and their overall sociodemographic status, 2016.

Study variables	Total	Stunting	Underweight	Wasting
**Sex of the child**		**< 0.001**	**0.021**	**0.667**
Male	4851	2003 (41.3%)	1199 (24.7%)	499 (10.3%)
Female	4643	1634 (35.2%)	1017 (21.9%)	460 (9.9%)
**Child age in months**		**< 0.001**	**< 0.001**	**< 0.001**
< 12 months	2061	321 (15.6%)	278 (13.5%)	298 (14.5%)
12–23 months	1896	780 (41.1%)	444 (23.4%)	239 (12.6%)
23–35 months	1788	869 (48.6%)	466 (26.1%)	168 (9.4%)
35–47 months	1820	857 (47.1%)	466 (25.6%)	124 (6.8%)
47–59 months	1929	810 (42.0%)	562 (29.1%)	131 (6.8%)
**Recent diarrhea**		**0.255**	**0.003**	**0.371**
No	8356	3171 (37.9%)	1884 (22.5%)	831 (9.9%)
Yes	1138	466 (40.9%)	332 (29.1%)	128 (11.2%)
**Recent acute respiratory infection**		**0.527**	**0.672**	**0.255**
No	7570	2883 (38.1%)	1756 (23.2%)	744 (9.8%)
Yes	1924	755 (39.2%)	460 (23.9%)	216 (11.2%)
**Number of children under 5 years**		**0.039**	**0.049**	**0.001**
1 child	3500	1323 (37.8%)	735 (21.0%)	308 (8.8%)
2 children	4324	1740 (40.2%)	1093 (25.3%)	415 (9.6%)
3 and above children	1670	574 (34.4%)	387 (23.2%)	237 (14.2%)
**Mother’s age (years)**		**0.469**	**0.827**	**0.581**
15–24	2104	810 (38.5%)	479 (22.8%)	221 (10.5%)
25–29	2898	1064 (36.7%)	650 (22.4%)	265 (9.1%)
30–34	2178	831 (38.2%)	528 (24.2%)	213 (9.8%)
35–39	1491	616 (41.3%)	358 (24.0%)	160 (10.7%)
40–49	823	317 (38.5%)	201 (24.4%)	100 (12.2%)
**Mother’s body mass index**		**< 0.001**	**< 0.001**	**0.001**
Underweight	1840	755 (41.0%)	554 (30.1%)	250 (13.6%)
Normal	6974	2703 (38.8%)	1580 (22.7%)	666 (9.5%)
Overweight	680	179 (26.3%)	82 (12.1%)	43 (6.3%)
**Mother’s educational status**		**< 0.001**	**< 0.001**	**0.0520**
No education	6225	2587 (41.6%)	1677 (26.9%)	677 (10.9%)
Primary	2600	919 (35.3%)	465 (17.9%)	234 (9.0%)
Secondary and above	669	130 (19.4%)	74 (11.1%)	49 (7.3%)
**Mother’s Occupation**		0.7307	0.4475	0.4377
Not working	6918	2639 (38.1%)	1637 (23.7%)	683 (9.9%)
Working	2576	999 (38.8%)	579 (22.5%)	277 (10.7%)
**Household wealth index**		**< 0.001**	**< 0.001**	**< 0.001**
Poorest	2188	986 (45.1%)	673 (30.8%)	304 (13.9%)
Poorer	2225	958 (43.1%)	593 (26.7%)	219 (9.8%)
Middle	2002	757 (37.8%)	457 (22.8%)	210 (10.5%)
Richer	1722	593 (34.4%)	293 (17.0%)	121 (7.0%)
Richest	1357	343 (25.3%)	200 (14.7%)	105 (7.7%)
**Major source of Water**		**0.652**	**0.649**	**0.783**
Unimproved	5325	2023 (38.0%)	1227 (23.0%)	545 (10.2%)
Improved	4169	1615 (38.7%)	989 (23.7%)	414 (9.9%)
**Type of toilet facility**		**< 0.001**	**< 0.001**	**0.002**
No facility	3471	1525 (43.9%)	1006 (29.0%)	421 (12.1%)
Unimproved	5205	1905 (36.6%)	1085 (20.8%)	457 (8.8%)
Improved	818	207 (25.3%)	125 (15.3%)	81 (9.9%)
**Place of residence**		**< 0.001**	**< 0.001**	**0.488**
Urban	1045	274 (26.2%)	147 (14.1%)	97 (9.3%)
Rural	8449	3364 (39.8%)	2069 (24.5%)	863 (10.2%)
**Regions in Ethiopia**		**< 0.001**	**< 0.001**	**< 0.001**
Tigray	647	252 (38.9%)	146 (22.6%)	73 (11.3%)
Amhara	1865	879 (47.1%)	535 (28.7%)	187 (10.0%)
Oromia	4164	1507 (36.2%)	921 (22.1%)	442 (10.6%)
SNNPR	1947	757 (38.9%)	409 (21.0%)	121 (6.2%)
Developing regions	605	191 (31.5%)	180 (29.8%)	122 (20.2%)
Addis Ababa, Dire Dawa and Harari	266	52 (19.5%)	25 (9.4%)	14 (5.3%)

### Prevalence of undernutrition

Seven out of fifteen children 46.5% [95% CI: 44.6, 48.4] have suffered from at least one form of malnutrition, while 3.1% [95% CI:2.6, 3.7] suffered from the three kinds of undernutrition (underweight, stunting and wasting). The prevalence of underweight was 23.3% [95% CI:21.9, 24.9], of these 16.8% [95% CI:15.7, 18.1] severe underweight. Three out of eight children (38.3%) [95% CI:36.4, 40.2] experienced stunting and 10.1% [95% CI:9.1, 11.2] wasting. Children were suffering from more than one form of undernutrition. Underweight and stunting in composes the highest share with 18.8%. The rest, 6.4% were suffering from wasting and underweight, and 3.1% wasting and Stunting (Table 2).

**Table 2:** Prevalence of undernutrition, Ethiopia Demographic and Health Survey (EDHS), 2016 (N = 9,464).

Types of undernutrition			Confidence interval (95%)	
	Frequency	Percent	Lower	Upper
**Stunting**				
No stunting	5857	61.7	59.8	63.6
Stunting	3637	38.3	36.4	40.2
Moderate stunting	1647	17.4	15.9	18.9
Severe stunting	1990	21.0	19.8	22.2
**Underweight**				
No underweight	7278	76.7	75.1	78.1
Underweight	2216	23.3	21.9	24.9
Moderate underweight	618	6.5	5.7	7.4
Severe underweight	1598	16.8	15.7	18.1
**Wasting**				
No wasting	8535	89.9	88.8	90.9
Wasting	959	10.1	9.1	11.2
Moderate wasting	282	3.0	2.4	3.6
Severe wasting	677	7.1	6.4	8.0
Stunting & Underweight	1789	18.8	17.5	20.3
Stunting & Wasting	292	3.1	2.6	3.7
Underweight & Wasting	609	6.4	5.6	7.3
**All forms of undernutrition**	292	3.1	2.6	3.7
**At least one form of undernutrition**	4414	46.5	44.6	48.4

### Determinants of undernutrition

Multiple factors were associated with underweight, stunting or wasting. Among the child characteristics, sex of a child, age of the child and recent experience of diarrhea associated with at least one form of undernutrition. Mother’s factors associated with undernutrition included body mass index and educational status. Household factors associated with undernutrition were household wealth index, place of residence, type of toilet facility and regions in Ethiopia. When taken to multivariate logistic regression, many of the variables were found to be significantly associated with undernutrition, as shown in Table 3.

**Table 3:** Factors associated with stunting, underweight, and wasting among under 5 children in Ethiopia, 2016.

Study variables	Stunting Adjusted OR (95% CI)			Underweight Adjusted OR (95% CI)			Wasting Adjusted OR (95% CI)		
**Sex of the child**									
Female	1.00 (Reference)			1.00 (Reference)					
Male	1.26 (1.16, 1.38) ***			1.17 (1.02, 1.36)*					
**Child age in months**									
< 12 months	1.00 (Reference)			1.00 (Reference)			1.00 (Reference)		
12–23 months	4.13 (3.10, 5.51) ***			1.89 (1.42, 2.50) ***			0.83 (0.62, 1.10)		
23–35 months	5.87 (4.47, 7.70) ***			2.27 (1.72, 3.00) ***			0.58 (0.43, 0.79) ***		
35–47 months	5.41 (4.14, 7.06) ***			2.25 (1.73, 2.92) ***			0.40 (0.28, 0.56) ***		
47–59 months	4.28 (3.23, 5.68) ***			2.70 (2.00, 3.64) ***			0.41 (0.29, 0.58) ***		
**Recent diarrhea**									
No	1.00 (Reference)			1.00 (Reference)			1.00 (Reference)		
Yes	1.30 (1.01, 1.66) *			1.63 (1.25, 2.13) ***			1.05 (0.75, 1.48)		
**Number of children under 5 years **									
One child	1.00 (Reference)			1.00 (Reference)			1.00 (Reference)		
Two children	1.02 (0.87, 1.21)			1.18 (0.95, 1.46)			1.08 (0.82, 1.43)		
3 and above children	0.89 (0.70, 1.14)			1.17 (0.85, 1.61)			1.51 (1.05, 2.18) *		
**Mother’s body mass index**									
Underweight	1.00 (Reference)			1.00 (Reference)			1.00 (Reference)		
Normal	0.89 (0.73, 1.09)			0.67 (0.54, 0.82) ***			0.69 (0.54, 0.89) **		
Overweight	0.78 (0.53, 1.13)			0.43 (0.27, 0.67) ***			0.48 (0.26, 0.92) *		
**Mother’s educational status**									
No education	1.00 (Reference)			1.00 (Reference)			1.00 (Reference)		
Primary	0.93 (0.77, 1.11)			0.72 (0.58, 0.90) **			0.88 (0.67, 1.17)		
Secondary and above	0.58 (0.42, 0.81) **			0.61 (0.41, 0.93) *			0.78 (0.49, 1.22)		
**Mother’s Occupation**									
Not working	1.00 (Reference)			1.00 (Reference)					
Working	1.04 (0.88, 1.23)			1.05 (0.88, 1.27)					
**Household wealth index**									
Poorest	1(Reference)			1.00 (Reference)			1.00 (Reference)		
Poorer	0.94 (0.71, 1.24)			0.94 (0.72, 1.22)			0.84 (0.58, 1.23)		
Middle	0.75 (0.55, 1.02)			0.79 (0.58, 1.05)			0.90 (0.60, 1.36)		
Richer	0.63 (0.46, 0.86) **			0.55 (0.40, 0.76) ***			0.64 (0.42, 0.99) *		
Richest	0.50 (0.34, 0.73) ***			0.71 (0.45, 1.14)			0.74 (0.43, 1.27)		
**Type of toilet facility**									
No facility	1.00 (Reference)			1.00 (Reference)			1.00 (Reference)		
Unimproved	0.91 (0.75, 1.10)			0.85 (0.68, 1.07)			0.91 (0.68, 1.22)		
Improved	0.83 (0.55, 1.27)			0.81 (0.51, 1.27)			1.16 (0.78, 1.73)		
**Place of residence**									
Urban	1.00 (Reference)			1.00 (Reference)					
Rural	0.93 (0.66, 1.33)			1.18 (0.79, 1.75)					
**Regions in Ethiopia**									
Addis Ababa, Dire Dawa and Harari	1.00 (Reference)			1.00 (Reference)			1.00 (Reference)		
Tigray	1.56 (1.08, 2.24) **			1.44 (0.95, 2.20)			1.78 (1.11, 2.85) **		
Amhara	2.10 (1.45, 3.04) ***			2.00 (1.27, 3.15) **			1.69 (1.00, 2.88) *		
Oromia	1.17 (0.79, 1.73)			1.33 (0.85, 2.09)			1.63 (0.97, 2.72)		
SNNPR^1^	1.36 (0.93, 1.98)			1.32 (0.85, 2.07)			1.03 (0.61, 1.75)		
Developing regions	0.95 (0.67, 1.34)			1.85 (1.22, 2.82) **			2.93 (1.82, 4.70) ***		

*** P-value <0.001

**P-value<0.01

*P-value<0.05

^1^ Southern nation, nationalities, and people’s region

### Factors associated with stunting

Sex of child [Male AOR = 1.26, 95% CI: 1.16, 1.38], child age [12–23 months (AOR = 4.13, 95% CI: 3.10, 5.51), (23–35 months AOR = 5.87, 95% CI: 4.47, 7.70), (36–47 months AOR = 5.41, 95% CI: 4.14, 7.06), and (47–59 months AOR = 4.28, 95% CI: 3.23, 5.68) compared to 0–12 months’ children]. Recent experience of diarrhea [AOR = 1.30, 95% CI: 1.01, 1.66], among regions [Tigray AOR = 1.56, 95% CI: 1.08, 2.24), and (Amhara AOR = 2.10, 95% CI: 1.45, 3.04), were more likely to be stunted compared with under-five children from Addis Ababa, Dire Dawa, and Harari.

On the other hand, protective factors included mother’s educational status [secondary and above AOR = 0.58, 95% CI: 0.42, 0.81 compared to children whose mother had no formal education], household wealth index [(richer AOR = 0.63, 95% CI: 0.46, 0.86), and (richest AOR = 0.50, 95% CI: 0.34, 0.73)] were less likely to be stunted compared with children from the poorest household.

### Factors associated with underweight

Sex of the child [Male AOR = 1.17, 95% CI: 1.02, 1.36) compared to female children], Age of the child between 12–23 months [AOR = 1.89, 95% CI: (1.42, 2.50)], 23–35 months [AOR = 2.27, 95% CI: 1.72, 3.00], 35–47 months [AOR = 2.25, 95% CI: 1.73, 2.92], and 47–59 months [AOR = 2.70, 95% CI: (2.00, 3.64)] compared, Recent experience of diarrhea [AOR = 1.63, 95% CI: (1.25, 2.13)], being from Amhara region [AOR = 2.00, 95% CI: 1.27, 3.15)], and Developing regions [AOR = 1.85, 95% CI: 1.22, 2.82] were significantly associated with underweight. On the contrary, underweight protective factors included mother’s educational status [Primary AOR = 0.72, 95% CI: 0.58, 0.90, Secondary and above AOR = 0.61, 95% CI: 0.41, 0.93] and household wealth index [richer 0.55, 95% CI: (0.40, 0.76),] were less likely to experience underweight.

### Factors associated with wasting

Number of under-five children in the house [3 and above AOR = 1.51, 95% CI: 1.05, 2.18], regions in Ethiopia [Tigray AOR = 1.78, 95% CI: 1.11, 2.85, Amhara AOR = 1.69, 95% CI: 1.00, 2.88, and developing regions AOR = 2.93, 95% CI: (1.82, 4.70)] were more likely to experience wasting compared to their counterparts.

On the other hand, child age [23–35 months AOR = 0.58, 95% CI: 0.43, 0.79, 35–47 months AOR = 0.40, 95% CI: 0.28, 0.56, 47–59 months AOR = 0.41, 95% CI: 0.29, 0.58] compared to 0–12 months’ children], Mother’s BMI [Normal AOR = 0.69, 95% CI: 0.54, 0.89, overweight AOR = 0.48, 95% CI: 0.26, 0.92] compared with children whose mother were underweight, household wealth index [richer AOR = 0.64, 95% CI: 0.42, 0.99] were less likely to experience wasting compared with children from poorest households (Table 3).

## Discussions

Overall, 46.5%(95% CI: 44.6, 48.4) of all children have suffered to the minimum from one form of undernutrition. Based on WHO prevalence thresholds and classification labels, the prevalence of stunting in this study falls in a very high category (38.3% (95%CI: 36.4, 40.2), and wasting in the medium division (10.1% (95%, CI: 9.1, 11.2) while underweight was in high (23.3% (95%CI: 21.9, 24.9)) [7].

Very high prevalence of stunting in this study is an indication of the significance of the problem in the sub-Saharan mainly Eastern African region; which was also reported from Rwanda, Zambia, Middle Africa, Oceania and Southern Asia [2,21–23]. Even though the burden of the stunting is still very high, there was an improvement to children affected by stunting. The global zero hunger target [8], which was also shared by the government of Ethiopia might have contributed to the observed improvements as compared to previous results of Ethiopian DHS[24–26]. Stunting in this study is also lower than that of the results from Burundi 57.5% [27], Malawi 47.1%, Mozambique 42.6%, and Democratic Republic of Congo 42.7%[28–31].

Even though the stunting during the five years preceding the survey period, the magnitude of stunting was higher than in most African countries. The results of DHS reports from Uganda 33.4%, Zimbabwe 32.0%, Lesotho 33.2%, Burkina Faso’s DHS (34.8%), had the lower stunting prevalence with current study[32–34]. It was also much higher than, Kenya 26.0%, Ghana 18.8%, and Togo 27.5%[35–37].

Wasting in the current study was lower than the reports of DHS of various countries like Burkina Faso 15.5%, Niger 18.0% and Chad 13.0%[38–40]. It was similar to the result of Sierra Leone 9.3%[41]. In contrary to this, it was higher than that of Nigeria 8.7%, Kenya 4.0%, Malawi 4.0%, Gabon 3.3% and Equatorial Guinea 3.1%[28,35,42–44]. Prevalence of underweight was higher than most African countries including, Zambia 1.5%, Rwanda 9.3%, Tanzania 13.7%. It was lower than the reports from Nigeria 28.7%, Chad 28.8%, Burundi 28.8%, and Niger 36.4%[21,22,40,44–46]. In general, this high prevalence of undernutrition in Ethiopia could be due to repeated attacks of drought and famine in the country. If the children suffer from starvation and undernutrition, then they will be exposed to decreased immunity and repeated infections [47,48].

Male children were 26 percent more likely to be affected by stunting as compared to their female counterparts. Similarly, the odds of male child suffering from underweight increased by 17 percent than the female child. Male children are more active physically than female children; thereby the energy utilization tends to be higher, which could have been used for healthy growth. Furthermore, other existing evidence also shows the high vulnerability of males from chronic infections which, in return, make them be at higher risk to the undernutrition. This finding is consistent with the reports from Ethiopia, Somalia, Tanzania and Nigeria [49–54], indicating that male children were more vulnerable to undersize and underweight.

The risk of stunting increased by four to six times as the age of the children exceeds 12 months. Similarly, older children were suffering from underweight as the odds of underweight risen by three to four times than under one-year-old children. However, this result was not consistent with wasting. Children whose age were 24 months and above were less likely to be exposed to wasting as compared to children under one year which could be due to the attention given to the children in infancy as compared to others. In Ethiopia, about 97 percent of the mothers feed their child breast milk nationally, with leading exclusive breastfeeding habit yet not enough [17], and this might have minimized the risk of undernutrition notably stunting and underweight during the first year of their age. These findings could also attribute to the challenges during the transition period to complementary feeding and weaning, as also supported by other studies [13,52,53,55].

Exposure to diarrhea lately before the survey was found to increase the odds of stunting and underweight. Those children who suffered from diarrhea of any cause were 30 and 63 percent more likely exposed to stunting and underweight as compared to those did not suffered from diarrhea, while it did not show association to wasting. This finding is also consistent with other studies[49,51,56]. The effect of diarrheal disorder is known in its potential to affect the nutritional status of children as it could cause dehydration, which may result in malnutrition, this, in turn, will expose children to new episodes of diarrhea due to decreased appetite and absorptions of the nutrients. So, the overall risk to undernutrition raised chronically.

Maternal factors associated with undernutrition include the nutritional status and education status. As reported in various studies, maternal nutritional status also predicted child undernutrition in this study [51,53]. The children whose mothers’ BMI were normal were 33 and 31 percent less exposed to underweight and wasting as compared to those children whose mothers’ with underweight BMI. On the other hand, children born to overweight mothers were found to be more protected from underweight and wasting. Maternal nutritional status during the antenatal period tends to affect the intrauterine growth of the fetus, which in return affects the latter nutritional status of the baby during the postnatal period.

As maternal educational status raises, the odds of developing stunting and underweight decreased. Mothers with a better educational background are believed to provide improved childcare and are more capable of handling nutritional and health issues of their children, while those with no education may not. Besides, women with advanced education will improve the household income and quality of life for the whole family more specifically to their children. This finding is in agreement with studies from Ethiopia, Tanzania, Nigeria and Bangladesh[50,53,57–60].

Presence of additional under-five children in the household might has raised the occurrences of wasting. Presence of three and above under-five children in the house increased the odds of wasting by half as compared to households having one under-five child. As the number of under-five children increases, the ability to fulfill the dietary need become a challenge to parents, this condition will worsen in developing countries like Ethiopia. As the number of children increases, the children’s competition will maximize as also evidenced by other studies [51,52,61]. Therefore, the exposure to less dietary intake could minimize the immunity of the children and then they will experience illness, from which they also suffer from wasting.

Besides, the odds of being stunted, wasted and underweight increased among children from less wealthy households, as also evidenced by various previous studies [53,58,62–64]. When it comes to the issue of food security, it is obvious that the poorest community or low-income countries are in bigger trouble. As we cannot separate the food security from nutritional status, children from the poorest family also live in poor nutritional status. If the children experience any additional health problem, child could be acute illness or chronic problem, they could not acquire immediate quality health care unlike those from high income households mainly because they could not afford the health care cost unless supported. Thereby the overall burden of undernutrition remains high among children born to poorest households.

The impact of undernutrition varied between the administrative regions of Ethiopia. To our knowledge, there were no previous studies identified the region as a predictor of under-nutrition. Agriculture is the primary source of economy in Ethiopia. Most of these regions depend on agriculture to make a living, but most of the regions are affected by frequent drought and food shortage. The variations of the drought among the regions might be contributed to the variations of undernutrition and remain as a great challenge to ensure food security.

We performed the analysis using multilevel logistic regression unlike previous studies, and this will make this finding more reliable than other similar studies conducted on undernutrition, furthermore involving individuals throughout the country also add strength. Even though the data were nationally representative, we did not include the types, and biochemical components of the diet children took, which may have contributed in identifying determinants related to dietary type.

## Conclusion

A considerable proportion of children suffer from undernutrition in Ethiopia. Based on this analysis, multiple factors associated with child undernutrition, child factors include Sex of child (male), Child age in months, and episodes of diarrhea. Mothers’ nutritional status and maternal education, were also associated with undermatron of their children. Poorest households’ wealth index was a factor associated with increased risk of undernutrition while the administrative region that the children reside was a community-level factor contributing to under-nutrition. Health promotion and prevention strategies under implementations should be continued by improving the nutritional status of children under five years of age to achieve the global nutrition target.

## Abbreviations

AOR: Adjusted Odds Ratios
BMI: Body Mass Index
CDC: Centers for Disease Control
CI: Confidence Interval
CSA: Central Statistical Agency
DHS: Demographic and Health Survey
EDHS: Ethiopia Demographic and Health Survey
NCHS: National Centers for Health Statistics
SNNPR: Southern Nation Nationalities and Peoples Region
SD: Standard Deviation
WAZ: weight for age Z score
HAZ: Height-for-Age Z score
WHZ: Weight-for-Height Z score
WHO: World Health Organization.
